# Comparison of 14 respiratory pathogens among hospitalized children during and after the COVID-19 outbreak in Chaoshan area

**DOI:** 10.1186/s12985-023-02040-z

**Published:** 2023-04-18

**Authors:** Chuang-Xing Lin, Hao-bin Lian, Guang-Yu Lin, Dan-gui Zhang, Xiao-Ying Cai, Fei-qiu Wen

**Affiliations:** 1 Department of Pediatrics, The Second Affiliated Hospital of Shantou University Medical College, Shantou, Guangdong China; 2 Translational Medicine Research Center, The Second Affiliated Hospital of Shantou University Medical College, Shantou, Guangdong China; 3 Shenzhen Children’s Hospital, Shenzhen, Guangdong China

**Keywords:** COVID-19, Epidemiology, Pediatrics, Respiratory infections, Children, Outbreak

## Abstract

**Background:**

Since January 2020, measures has been adopted in the Chaoshan area to limit the spread of COVID-19. Restrictions were removed after August 2020. At the same time, children returned to school. We previously reported the changes of 14 main respiratory pathogens in hospitalized children before and during the COVID-19 outbreak in Chaoshan area. However, the changes of respiratory pathogen spectrum in hospitalized children after the epidemic are still unknown, which will be elucidated in this study.

**Methods:**

There are 6201 children hospitalized with respiratory tract infection were enrolled in the study, which were divided into two groups: 2533 from outbreak group (1 January 2020–31 December 2020), and 3668 from post-outbreak group (1 January 2021–31 December 2021). Pharyngeal swab samples were collected. 14 respiratory tract pathogens were detected by liquid chip technology.

**Results:**

The positive rate of pathogen detection is significantly lower in the outbreak group (65.42%, 1657/2533) than that in the post-outbreak group (70.39%, 2582/3668; χ^2^ = 17.15, *P* < 0.05). The Influenza A virus (FluA) detection rate was 1.9% (49) in 2020, but 0% (0) in 2021. The detection rates of *Bordetella pertussis* (BP) decreased from 1.4% (35) in 2020 to 0.5% (17) in 2021. In contrast, the detection rates of  *Influenza B virus* (FluB), *Cytomegalovirus* (CMV), *Haemophilus influenzae* (HI), *Streptococcus pneumoniae* (SP) increased from 0.3% (8), 24.7% (626), 2.0% (50) and 19.4% (491) in 2020 to 3.3% (121), 27.9% (1025), 4.6% (169), 22.8% (836) in 2021, respectively (*P* < 0.01).

**Conclusions:**

The detection rates of pathogens such as FluA, FluB, CMV, HI, SP, BP were statistically different between 2020 and 2021. From 2020 to 2021, the positive rates of Flu, CMV, HI and SP increased, while the positive rates of FluA and BP decreased. After the COVID-19 prevention and control measures are gradually relaxed, the positive rate of respiratory pathogens in children aged from 6 months to 6 years will increase.

## Background

At the end of 2019, an outbreak of COVID-19 occurred in Wuhan, Hubei Province, China, and it rapidly swept the country. The Chinese government rapidly adopted a series of prevention and control measures. Under this condition, from January 2020 to September 2020, in the Chaoshan area measures were also adopted to limit the spread of COVID-19, such as reducing population aggregation, wearing masks in public and suspending classes. In our previous study [1], we found that: firstly, during the COVID-19 outbreak, the number of children hospitalized for respiratory infection was significantly reduced; secondly, the overall positive rate of respiratory pathogens decreased obviously, and last, the proportion of bacterial infection decreased, while the proportion of viral infection did not decrease significantly [1]. Similarly, a recent study on Shenzhen City demonstrated that outbreak control measures have resulted in significantly lower pathogen detection rates among children hospitalized with respiratory infections, especially for influenza [2]. However, the detection rates of *respiratory syncytial virus (RSV), parainfluenza virus (PIV), and Human metapneumovirus (hMPV)* increased significantly from September to December 2020 (during the COVID-19 epidemic) in Shenzhen [2]. However, it is still unclear whether the spectrum of 14 respiratory pathogens would change in hospitalized children after the epidemic. This study aims to shed light on which pathogens will be altered after prevention/control measures during COVID-19 outbreak.

## Materials and methods

### Patients and ethics statement

In this study, we collected 6201 pharyngeal swabs from children hospitalized with respiratory tract infections in Pediatrics department of the Second Affiliated Hospital of Shantou University Medical College, which is the largest children’s medical center in Chaoshan area, from 1 January 2020 to 31 December 2021. The children ranged in age from 1 month to 14 years. The inclusion and exclusion criteria were the same as those mentioned in our previous study [1]. Using January 1, 2021 as the boundary, children enrolled in the study were divided into two groups (Table 1): Outbreak group (1 January 2020–31 December 2020), and Post-outbreak group (1 January 2021–31 December 2021). In outbreak group (Year 2020), the mean age was 2 years and 2 months, while the median age was 1 year. In post-outbreak group (Year 2021), the mean age was 2 years and 5 months, while the median age was 1 year. In both the outbreak and post-outbreak groups, the ratio of boys to girls was 1.5–1. According to age, we divided the patients into five groups as follows: (1) 0 to ≤ 6 months, 621 cases in 2020 and 755 cases in 2021; (2) > 6 months to ≤ 1 year, 839 cases in 2020 and 1099 cases in 2021; (3) > 1 year to ≤ 3 years, 322 cases in 2020 and 961 cases in 2021; (4) > 3 years to ≤ 6 years, 267 cases in 2020 and 552 cases in 2021; (5) > 6 years, 174 cases in 2020 and 301 cases in 2021. Study protocol was approved by the ethics committee of the Second Affiliated Hospital of Shantou University Medical College. Written informed consent was obtained from the children’s parents.

**Table 1:** Comparison of positive rates of 14 respiratory pathogens between Year 2020 and Year 2021

Pathogen	Year 2020 (%) N = 2533	Year 2021(%) N = 3668	χ^2^	*P* (0.05)
FluA*	49 (1.9)	0 (0.0)	71.521	0.000
FluB*	8 (0.3)	121 (3.3)	65.449	0.000
PIV	167 (6.6)	279 (7.6)	2.305	0.129
HRV	304 (12.0)	445 (12.1)	0.024	0.877
ADV	13 (0.5)	10 (0.3)	2.347	0.126
RSV	468 (18.5)	735 (20.0)	2.338	0.126
hMPV	47 (1.9)	60 (1.6)	0.427	0.514
hBoV	38 (1.5)	80 (2.2)	3.720	0.054
CMV*	626 (24.7)	1025 (27.9)	8.005	0.005
HI*	50 (2.0)	169 (4.6)	30.500	0.000
SP*	491 (19.4)	836 (22.8)	10.343	0.001
MC	126 (5.0)	213 (5.8)	2.010	0.156
MP	28 (1.1)	50 (1.4)	0.801	0.371
BP*	35 (1.4)	17 (0.5)	15.194	0.000

Pathogens labelled (*) showed significant differences between different years

### Specimen collection and pathogen detection

The pharyngeal swabs were collected by trained doctors or nurses according to standard operating procedures within 24 h after admission. The specimens were sent to Guangzhou Da’an Clinical Examination Center. The total nucleic acids were extracted by using the viral total nucleic acid extraction kit (Magen Biotechnology, Guangzhou, China), according to the manufacturers’ instructions. Luminex liquid chip technology [3] and multiplex polymerase chain reaction (PCR) [4] were used to detect nucleic acids of respiratory pathogens. In this way, 14 main respiratory pathogens can be detected at the same time, including *influenza A virus (FluA), influenza B virus (FluB), parainfluenza virus (PIV), respiratory syncytial virus (RSV), human rhinovirus (HRV), cytomegalovirus (CMV), H. metapneumovirus (hMPV), bocavirus (BoV), adenovirus (ADV), Haemophilus influenzae (HI), Mycoplasma pneumoniae (MP), Streptococcus pneumoniae (SP), Moraxella catarrhalis (MC), and Bordetella pertussis (BP).*

### Statistical methods

SPSS *v*23 was used to analyze data. Chi-square test [5] or Fisher’s exact test [6] was used to compare the positive detection rate of each pathogen among three groups. *P* value less than 0.05 was considered statistically significant.

## Results

### Basic information

The positive rate of pathogen detection is significantly lower in the outbreak group (65.42%, 1657/2533) than that in the post-outbreak group (70.39%, 2582/3668; χ^2^ = 17.15, *P* < 0.05).

### Changes of specific pathogens between 2020 and 2021

The top five pathogens detected in the outbreak and the post-outbreak groups were the same. They were CMV, SP, RSV, HRV and PIV. The positive rates of detection of FluA, FluB, CMV, Hi, SP and BP significantly differed between the post-outbreak and outbreak groups (χ^2^ = 71.521, 65.449, 8.005, 30.500, 10.343 and 15.194 respectively; *P* < 0.05 for all, Table 1). However, the detection rates of other pathogens, such as PIV, HRV, ADV, RSV, hMPV, hBoV, MC and MP, didn’t significantly differ between the two groups (*P* > 0.05 for all, Table 1). None of even one case of FluA was detected in post-outbreak group, while there were 49 cases (4.0%) of FluA in outbreak group. The detection rates of *B. pertussis* (BP) decreased from 1.4% (35) in 2020 to 0.5% (17) in 2021. In contrast, the detection rates of influenza B virus (FluB), cytomegalovirus (CMV), *H. influenzae* (HI), *S. pneumoniae* (SP) increased from 0.3% (8), 24.7% (626), 2.0% (50) and 19.4% (491) in 2020 to 3.3% (121), 27.9% (1025), 4.6% (169), 22.8% (836) in 2021, respectively (*P* < 0.01).

### Variations in respiratory pathogens based on month and age

**Fig. 1:**
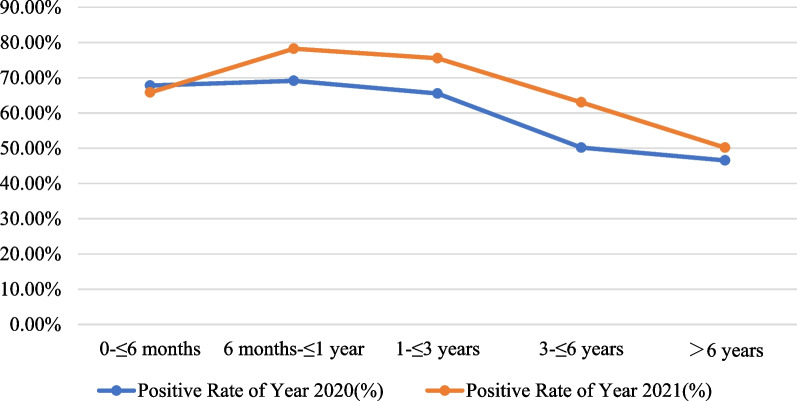
Comparison of pathogen positive rates by age groups between 2020 and 2021

**Fig. 2:**
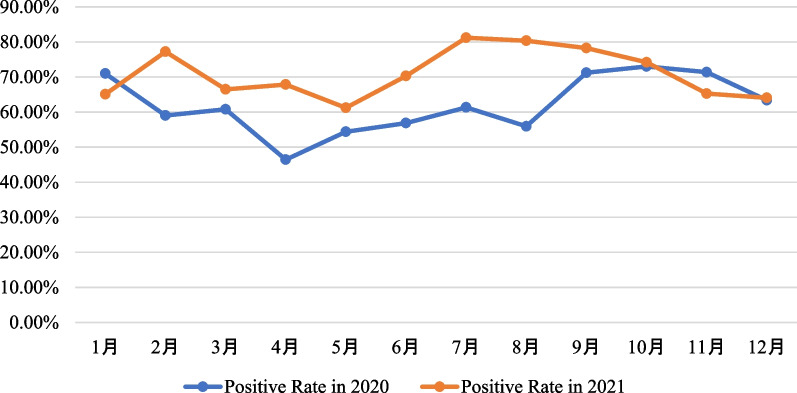
Comparison of pathogen positive rates by month between 2020 and 2021

Children aged 6 months to 6 years (> 6 months- ≤ 1 year; > 1 year- ≤ 3 years; > 3 years- ≤ 6 years) had significantly higher positive rates of respiratory pathogens in 2021 compared to 2020 (*P* < 0.05, Fig. 1). However, there was no significant difference in positive rates of respiratory pathogens between 2020 and 2021 for the other age groups (*P* > 0.05, Fig. 1). The differences in pathogen detection rates in February, April and June to September are statistically significant when comparing 2020 and 2021 (*P* < 0.05, Fig. 2).

**Fig. 3:**
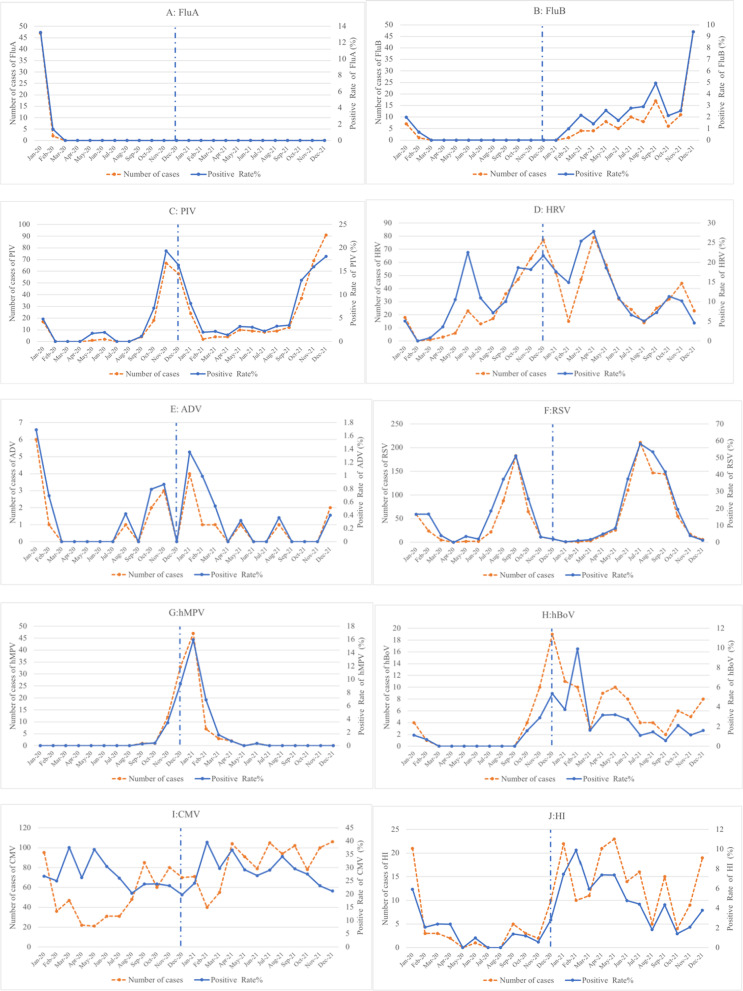

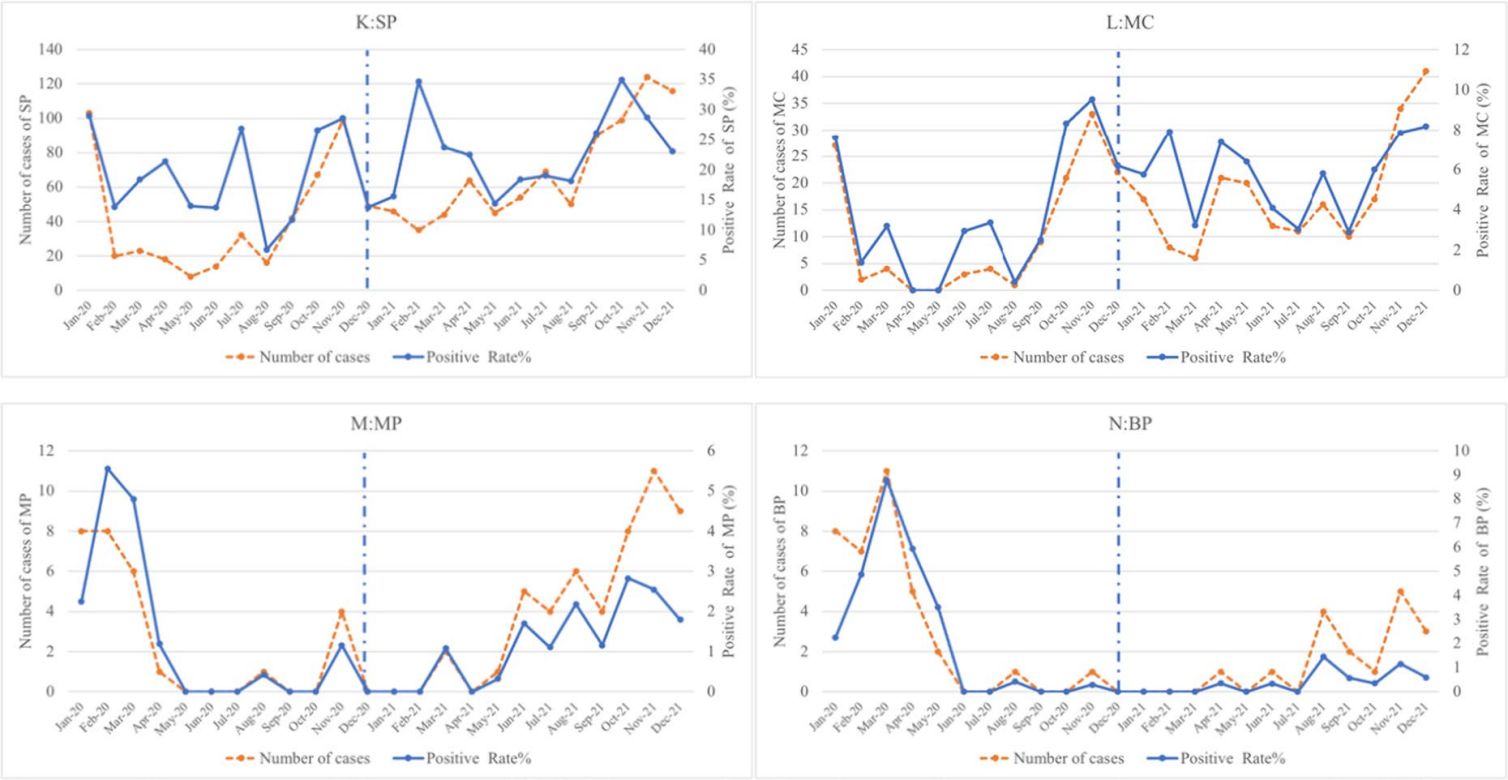
Variations in the detection of respiratory pathogens based on month. **A** FluA; **B** FluB; **C** PIV; **D** HRV; **E** ADV; **F** RSV; **G** hMPV; **H** BoV; **I** CMV; **J** Hi; **K** SP; **L** MC; **M** MP; **N**: BP

As shown in Fig. 3, FluA peaked in January 2020, declined rapidly in February 2020 and no further cases of FluA were detected from March 2020 to December 2021 (Fig. 3A). Only a few cases of FluB have been reported in January and February 2020 (Fig. 3B). However, in 2021, FluB shows a spiral increase and peaks in December 2021 (Fig. 3B).

The number of cases and detections of PIV increased rapidly in October 2020, peaked in November and December, and declined rapidly thereafter (Fig. 3C). PIV continued to increase from October to December 2021(Fig. 3C).

The number of HRV detections shows a downward trend from January to February 2020, with a small peak in June and a roughly monthly upward trend in the other months, reaching a large peak in December 2020 (Fig. 3D). Similarly, the detection rate of HRV also peaks in June and December 2020. The number of HRV cases shows a downward trend from January to February in 2021, with a peak in April, and then a monthly decline until August, when it starts to rise monthly, with a small peak in November (Fig. 3D). Similarly, the detection rate of HRV peaks in April and October 2021.

The number of cases of ADV peaks in January 2020, but only 6 cases. In the other months of 2020 and throughout 2021, only a few cases were detected or not detected (Fig. 3E).

The number of RSV detections declined from January to April 2020, began to rise rapidly in July, peaked in September, and declined rapidly thereafter (Fig. 3F). The number of RSV detections began to increase in May 2021, increased rapidly in June, peaked in July, and declined rapidly thereafter (Fig. 3F). The trend in RSV detection rates is generally consistent with the trend in the number of cases detected.

The number of cases and detection rate of hMPV showed an increase in November 2020 and a rapid increase in December, peaking in January 2021 and declining rapidly thereafter (Fig. 3G).

Only a few cases of BoV were reported in January and February 2020, with no detections from March to September. The number of BoV cases began to increase in October 2020, peaking in December, and declining thereafter, with small peaks in May, October and December 2021 (Fig. 3H). The detection rate of BoV peaks in December 2020 and February 2021 (Fig. 3H).

The number of cases of CMV will generally decline and then increase in 2020, with a small peak in March and a peak in September. the number of cases of CMV will reach a low point in February 2021, after which the number of cases will increase and fluctuate at relatively high levels (Fig. 3I). There is no clear pattern in the trend of CMV detection rates.

The number of HI detections peaks in January 2020, fluctuates at low levels each month thereafter, rises in December 2020, and peaks in January 2021 and fluctuates thereafter (Fig. 3J).The trend in HI detection rate and number of detections is broadly similar.

The number of SP detections will broadly follow a downward and then upward trend in 2020, with peaks in January and November, dropping to a low point in February 2021, and then broadly spiralling thereafter (Fig. 3K). There was no clear pattern in the detection rate of SP.

The number of detected cases of MC follows a general trend of decreasing and then increasing, with a peak in January 2020, fluctuating at low levels for several months thereafter, beginning to increase month by month in August, with a peak in November, a low point in March 2021, and fluctuating up and down thereafter, with another monthly increase in September 2021 and a peak in December 2021 (Fig. 3L). The trend in the detection rate of MC was similar to the trend in the number of cases detected.

The number of MP cases peaks in January and February 2020, declines in March and April, with only sporadic or no reports per month thereafter, and a spiralling increase starting in May 2021, with a peak in November (Fig. 3M). The detection rate of MP peaks in February 2020 and October 2021.

The number of BP cases peaked in March 2020, declined monthly in April and May, and was not detected or only a few cases were detected each month thereafter (Fig. 3N). The detection rate for BP peaks in March 2020.

## Discussion

Our study showed that the positive rate of 14 respiratory pathogens in children in Chaoshan region in 2021, the year after the relaxation of epidemic prevention and control measures was significantly higher than that in 2020, the epidemic year (70.39% in 2021 vs 65.42% in 2020, χ^2^ = 17.15, *P* < 0.05). The positive detection rates of 14 major respiratory pathogens among hospitalized children were similar to those described in other studies (from 60.3 to 85.2%) [7–9]. Most studies have shown that the positive rate of respiratory pathogens in children during the epidemic is significantly lower than that before the epidemic, indicating that the epidemic prevention measures against the novel coronavirus have also reduced the prevalence of respiratory pathogens [10–13]. However, this study found that once the above measures were removed, the positive rate of most respiratory pathogens in children would rise again.

Studies have shown that COVID-19 prevention and control measures can reduce *S. pneumoniae* infections. A multicenter, large-sample study published in the Lancet Digital Health showed that all participating countries and territories confirmed a significant reduction in invasive disease caused by *S. pneumoniae* infections in early 2020 (1 January–31 May 2020), which the researchers attributed to the measures taken by countries to combat COVID-19 [14]. In this study, the positive rate of SP in 2020 was 19.4% and 22.8% in 2021. There was a statistical difference in the comparisons of the SP positive rates in 2020 and 2021(χ^2^ = 10.343, *P* = 0.001). Although the epidemic prevention and control measures in 2020 will reduce the spread of SP to a certain extent, the detection rate of SP in 2020 is still high. In addition, after the gradual liberalization of epidemic control measures in 2021, the detection rate of SP was higher. Considering the high carrier rate of SP in healthy children in China (21.4%, 95% CI 18.3–24.4%) [13], we should pay attention to the vaccination of SP. Because many studies have confirmed that vaccines play an important role in the prevention of SP-related diseases [13, 15–17].

The detection rates of hMPV had no difference between outbreak group (2020) and post-outbreak group (2021) (χ^2^ = 0.427, *P* = 0.514, Table 1). The number and detection rate of hMPV began to increase from October 2020, reached the peak in January 2021, and then decreased month by month until it was 0 in May 2021. A study from the Netherlands observed a local peak of hMPV infection in June and July 2021 [18]. However, in previous years, the peak of hMPV infection in the region has been in March. Moreover, this peak in hMPV occurred just after the lifting of the national closure measures due to COVID-19. In this study, the peak of hMPV occurs in January 2021, also after the gradual relaxation of outbreak control measures. Therefore, public health measures may be key to influencing the spread and prevalence of hMPV.

FluA was at its peak in January 2020, probably because the outbreak prevention and control measures were just starting to be implemented and had not yet taken effect. However, FluA declined rapidly in February 2020 and no cases of FluA were detected from March 2020 to December 2021. This suggests that outbreak control measures have been effective in limiting the spread and prevalence of FluA. This effect can be maintained for a longer period of time. Only a few cases of FluB were detected in January and February 2020, and no case of FluB was detected from March to December 2020. However, in 2021, FluB shows a spiral increase and peaks in December 2021. A study from Sichuan Province, China, showed that the prevalence of influenza A decreased from 22.5% in 2019 to 9.9% in 2020 to 0.2% in 2021 (*P* < 0.001) [19]. On the contrary, the lowest and highest positive rates of FluB occurred in 2020 and 2021, respectively, and the comparison between the 3 years was statistically significant (*P* < 0.001) [19]. Therefore, we should pay attention to the changing trend of FluA in the future and be alert to the rebound of FluA. The change trend of BP detection rate in 2020 and 2021 was similar to that of FluA. The trend of HI detection rates in 2020 and 2021 was similar to that of FluB.

From April to July 2020, the number of CMV detected cases remained at a relatively low level, but the change trend of the number and detection rate of CMV in the other months of 2020 was similar to that in the corresponding months of 2021. Besides, the detection rates of CMV increased from 24.7% (626) in 2020 to 27.9% (1025) in 2021(*P* < 0.01). We also found that CMV was the most frequently detected of the 14 respiratory pathogens in both 2020 and 2021. It is well documented that CMV infection rates in the population are as high as 50–90%, but most people are latently infected or chronically infected without obvious clinical symptoms [20]. Therefore, epidemic prevention and control measures have little effect on the spread and prevalence of CMV.

Other pathogens, such as PIV, HRV, ADV, RSV, hBoV, MC and MP, showed no significant difference between 2020 and 2021. So, we're not going to talk about them all. In general, COVID-19 prevention and control measures had little effect on the prevalence of these pathogens.

In the study, the positive rate of respiratory pathogens in children aged 6 months–6 years (> 6 months to ≤ 6 years) in 2021 was significantly higher than that in 2020 (*P* < 0.05, Fig. 1). There was no significant difference in the positive rate of respiratory pathogens between 2020 and 2021 in the other age groups (*P* > 0.05, Fig. 1). We speculate that the reason may be that children younger than 6 months of age are protected by maternal IgG antibodies, whereas children older than 6 years of age have sufficient immunity. Therefore, there was no statistically significant difference in the positive rate of pathogens between these two age groups in 2020 and 2021. In contrast, children between 6 months and 6 years of age had a loss of protection from maternal IgG and insufficient autoimmunity, which resulted in a higher prevalence of pathogens in 2021 than in 2020.

February, April and June to September in 2021 have a higher rate of pathogen positivity than the same months in 2020. This phenomenon is very interesting, but the cause is currently unknown and requires further research.

Limitations: Data from the year before the pandemic were missing in this study, so a comparison between pre-pandemic, during pandemic, and post-pandemic could not be made. Besides, children enrolled in our study were all hospitalized, which might bring selection bias [21].

At present, the prevention and control measures against the COVID-19 epidemic in China have been fully liberalized. Our study may help to judge the changing trend of respiratory pathogen spectrum of children in Chaoshan area in the future, and deepen our understanding of 14 respiratory pathogens.

## Conclusion

The detection rates of pathogens such as FluA, FluB, CMV, HI, SP, BP were statistically different between 2020 and 2021. From 2020 to 2021, the positive rates of Flu, CMV, HI and SP increased, while the positive rates of FluA and BP decreased. After the COVID-19 prevention and control measures are gradually relaxed, the positive rate of respiratory pathogens in children aged from 6 months to 6 years will increase.

## Data Availability

All data generated and/or analyzed during this study were included in this published article.

## Abbreviations

FluA: *Influenza A virus*
FluB: *Influenza B virus*
PIV: *Parainfluenza virus*
RSV: *Respiratory syncytial virus*
HRV: *Human rhinovirus*
CMV: *Cytomegalovirus*
hMPV: *Human metapneumovirus*
BoV: *Bocavirus*
ADV: *Adenovirus*
HI: *Haemophilus influenza*
MP: *Mycoplasma pneumonia*
SP: *Streptococcus pneumonia*
MC: *Moraxella catarrhalis*
BP: *Bordetella pertussis*

## References

[1] Lin C-X, Lian H-B, Lin G-Y, Zhang D-G, Cai X-Y, Cai Z-W (2021). Pathogen spectrum changes of respiratory tract infections in children in Chaoshan area under the influence of COVID-19. Epidemiol Infect.

[2] Li L, Wang H, Liu A, Wang R, Zhi T, Zheng Y (2021). Comparison of 11 respiratory pathogens among hospitalized children before and during the COVID-19 epidemic in Shenzhen, China. Virol J.

[3] Yan Y, Luo JY, Chen Y, Wang HH, Zhu GY, He PY (2017). A multiplex liquid-chip assay based on Luminex xMAP technology for simultaneous detection of six common respiratory viruses. Oncotarget.

[4] Huang HS, Tsai CL, Chang J, Hsu TC, Lin S, Lee CC (2018). Multiplex PCR system for the rapid diagnosis of respiratory virus infection: systematic review and meta-analysis. Clin Microbiol Infect: Off Publ Eur Soc Clin Microbiol Infect Dis.

[5] Mchugh ML (2013). The chi-square test of independence. Biochem Med.

[6] Jung SH (2014). Stratified Fisher's exact test and its sample size calculation. Biomet J Biometrische Zeitschrift.

[7] Cantais A, Mory O, Pillet S, Verhoeven PO, Bonneau J, Patural H (2014). Epidemiology and microbiological investigations of community-acquired pneumonia in children admitted at the emergency department of a university hospital. J Clin Virol: Off Publ Pan Am Soc Clin Virol.

[8] Aktürk H, Sütçü M, Badur S, Törün SH, Çıtak A, Erol OB (2015). Evaluation of epidemiological and clinical features of influenza and other respiratory viruses. Turk Pediatri Arsivi.

[9] Zhong P, Zhang H, Chen X, Lv F (2019). Clinical characteristics of the lower respiratory tract infection caused by a single infection or coinfection of the human parainfluenza virus in children. J Med Virol.

[10] Edwards KM (2021). The impact of social distancing for severe acute respiratory syndrome coronavirus 2 on respiratory syncytial virus and influenza burden. Clin Infect Dis.

[11] Achangwa C, Park H, Ryu S, Lee MS (2022). Collateral impact of public health and social measures on respiratory virus activity during the COVID-19 pandemic 2020–2021. Viruses.

[12] Kyu-Bin Oh, Mark Doherty T, Vetter V, Bonanni P (2022). Lifting non-pharmaceutical interventions following the COVID-19 pandemic: the quiet before the storm?. Expert Rev Vaccin.

[13] Wang L, Fu J, Liang Z, Chen J (2017). Prevalence and serotype distribution of nasopharyngeal carriage of *Streptococcus*
*pneumoniae* in China: a meta-analysis. BMC Infect Dis.

[14] Brueggemann AB, Jansen van Rensburg MJ, Shaw D (2021). Changes in the incidence of invasive disease due to *Streptococcus*
*pneumoniae*, *Haemophilus*
*influenzae*, and *Neisseria*
*meningitidis* during the COVID-19 pandemic in 26 countries and territories in the Invasive respiratory infection surveillance initiative: a prospective analysis of surveillance data [published correction appears in Lancet Digit Health. 2021 May 26]. Lancet Digit Health.

[15] Isaacman DJ, McIntosh ED, Reinert RR (2010). Burden of invasive pneumococcal disease and serotype distribution among *Streptococcus*
*pneumoniae* isolates in young children in Europe: impact of the 7-valent pneumococcal conjugate vaccine and considerations for future conjugate vaccines. Int J Infect Dis: IJID: Off Publ Int Soc Infect Dis.

[16] Brady MT, Byington CL, Dele Davies H, Edwards KM, Jackson MA, Maldonado YA (2014). Immunization for *Streptococcus*
*pneumoniae* infections in high-risk children. Pediatrics.

[17] Lewnard JA, Givon-Lavi N, Dagan R (2021). Effectiveness of pneumococcal conjugate vaccines against community-acquired alveolar pneumonia attributable to vaccine-serotype *Streptococcus*
*pneumoniae* among children. Clin Infect Dis: Off Publ Infect Dis Soc Am.

[18] Kivit C, Groen K, Jongbloed M, Linssen C, van Loo A, van Gorp E, van Nieuwkoop S, den Hoogen BV, Kruif M (2022). An off-season outbreak of *Human*
*metapneumovirus* infections after ending of a COVID-19 lockdown. J Infect.

[19] Wang P, Xu Y, Su Z, Xie C (2023). Impact of COVID-19 pandemic on influenza virus prevalence in children in Sichuan, China. J Med Virol.

[20] Nikolich-Žugich J, Čicin-Šain L, Collins-McMillen D, Jackson S, Oxenius A, Sinclair J, Snyder C, Wills M, Lemmermann N (2020). Advances in cytomegalovirus (CMV) biology and its relationship to health, diseases, and aging. GeroScience.

[21] Tripepi G, Jager KJ, Dekker FW, Zoccali C (2010). Selection bias and information bias in clinical research. Nephron Clin Pract.

